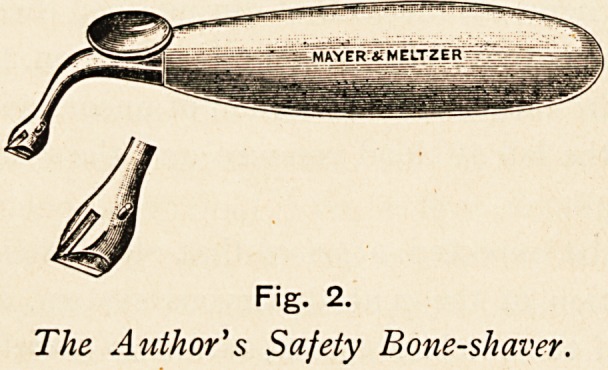# Some Points in the Technique of the Radical Operation for Chronic Otitis Media Purulenta
^1^Read at a meeting of the Bristol Medico-Chirurgical Society, April 14th, 1909.


**Published:** 1909-09

**Authors:** P. Watson Williams

**Affiliations:** Lecturer on Diseases of the Nose and Throat, University College, Bristol; Rhinologist and Laryngologist, Bristol Royal Infirmary


### SOME POINTS IN THE TECHNIQUE OF THE RADICAL OPERATION FOR CHRONIC OTITIS MEDIA PURULENTA.[1]

BY

P. WATSON WILLIAMS, M.D. Lond.,

*Lecturer on Diseases of the Nose and Throat, University College, Bristol; Rhinologist and Laryngologist, Bristol Royal Infirmary.*

---

To discuss the general principles and indications for the radical mastoid operation would be futile, as these are well recognised and established; but an operation which depends so largely on matters of detail for ultimate complete success allows for useful discussion on various subsidiary yet nevertheless important points, a few of which I propose to touch on.

[1] Read at a meeting of the Bristol Medico-Chirurgical Society, April 14th, 1909.

*Incision.*— Provided there is no special and complicating condition, such as a fistulous opening in the skin over the mastoid, there are certain advantages in an incision extending from a point half an inch above the highest point of the auricular attachment, curving backwards to about an inch and a half from the centre of the posterior meatal wall, and then forwards to a point just above the mastoid tip. Such an incision lies close to, or just within the hair line. The advantages are (1) it allows of exposure of the whole bone operative field, but lies well behind the bone excavation area ; (2) that no disturbance or retraction of the skin or periosteum behind the line of incision is required ; (3) better apposition and union is obtained from the tips of the wound lying on the undisturbed bone than when the incision lies over the excavated area, which has the double disadvantage of increasing the liability of wound infection from the aural cavity, and of allowing the sutured edges to sink and invert into the bone cavity. These may seem to be relatively unimportant points, but in practice the advantages become very apparent. Quite recently, in operating with Drs. Berry and Kelly for subacute mastoiditis in a child aged two years, a minute softened area of bone, about to become a fistula, was found after careful examination close to the outer posterior border of the large bone area thus exposed, leading down to purulent mastoid cells, thus greatly facilitating the operation. It is true that as the mastoid had to be opened out and cleared to an unusual extent, backwards and right down to the tip, leaving a very large cavity, the softened area would have eventually been reached in this case. Nevertheless, with usual anatomical conditions, with the more customary incision close to the post-auricular sulcus, the softened area would have been liable to remain undiscovered beneath the skin and soft tissues, and, anyhow, would have necessitated raising and retraction of the posterior flap to an unusual extent. As it was, even with these exceptional difficulties, the skin and tissues posterior to the incision were never raised or disturbed.

On the other hand, the more forward incision, a third of an inch only behind the line of the post-auricular groove, has advantages if for any reason the post-auricular incision is not to be sutured immediately on the completion of the operation, or if the bone cavity is to be opened up by a secondary operation for the purpose of a secondary skin grafting, a procedure which has largely fallen into disuse.

Contra-indications to the posterior incision advocated would be the existence of a fistula, as the line of incision would then be through the fistula; and in infants, before the development of the mastoid, care should be observed not to prolong the incision down below the level of the meatal floor.

Then the incision is usually carried down to the bone, except above, where it corresponds with the temporal muscle; but some operators carry the first incision only through the skin and soft tissues, and divide the periosteum after the skin and soft tissues have been raised and the flap carried forward. But, anyhow, it is usual to incise the periosteum posteriorly through the line of the first incision, and carry it forwards with the auricular flap, and backwards with the posterior flap.

Now I have often found it an advantage to separate the skin and soft tissues of the auricular flap as far as the posterior meatal margin, and there incise the periosteum, which is retracted and held back to the posterior tip of the curvilinear incision until the operation is completed, and then the periosteum is used as far as possible to cover the inner and back wall of the bony excavation. I generally make a Korner meatal flap, and when this is anchored up to the deep surface of the semilunar or auricular flap, it suffices to protect that surface and start epithelisation there; and the periosteum is not so useful there, between the Korner meatal skin flaps, as it is when laid on the posterior and deep wall of the bone cavity, to which it quickly unites, as it is kept in position by the gauze packing. Healthy granulations more rapidly spring up on the surface of the periosteum than on the bare bone, and thus the more rapid epithelisation of the bone cavity is promoted.

This method of utilising the periosteum corresponding with the area of bone which has been excavated is contra-indicated when the post-auricular tissues are boggy and inflamed and the integuments swollen, and more especially if pus is found there.

The next point I propose to touch on concerns the inspection and treatment of the inner tympanic wall, after the antrum has been found and, together with all infected mastoid cells, cleared and well laid open, and the bridge over the aditus has been removed, leaving an upper and lower pillar to the opened aditus. It is hardly possible to obtain a proper inspection of the inner tympanic wall without a good reflected light, for we have to note the condition of the lining membrane; the presence of granulations, more particularly in the region of the sinus tympanicus and hypotympanum; to examine the facial aqueduct for any defect in its wall, and note the external arcuate eminence; and examine for any point of erosion of the surface or opening into the lumen of the external semicircular canal. Granulations on the inner tympanic wall must be carefully curetted, avoiding, of course, disturbance of the stapes footplate, but the less we have to curette or injure the mucosa the better, since even infected mucous membrane may recover its normal healthy condition after obliteration of the source of infective pus, whereas extensive destruction of the mucous membrane leads to a free growth of healthy granulations in the process of healing, with consequent much greater diminution in hearing power when the blanket of granulations becomes covered with epithelium. It is quite possible to leave the patient with very useful hearing if we can get rid of all suppuration, and get the wound to heal without destroying the tympanic inner wall mucosa.

For instance, in a lady, aged 52, on whom I performed a radical mastoid operation in May, 1908, the hearing power in September for distinguishing words was 22 feet loud voice, 4 feet ordinary voice; and in the case I have shown to-night, on whom the radical operation was performed on December 11th, 1908, the hearing power to-day is for distinguishing words, 25 feet loud voice, 7 feet loud whisper, 10 inches the watch.

In neither case can it be urged that they were subacute cases, for in the first patient the aural suppuration had existed for forty years, and in the second patient for eighteen years. But, of course, it depends on the condition of the structures forming the internal tympanic wall whether or not such pleasing results are possible, and such conditions can be observed only by means of a thorough illumination by reflected light.

In practice it is generally better to reserve the examination of many points of detail till granulations in the hypotympanum are removed, as they bleed freely, but it is quite possible to overlook their presence if the hypotympanum space is at all deep ; in other words, if it has a well-marked external wall. Yet to leave them is to court failure, for particularly in the posterior portion there are often numerous small pneumatic cells which will be found suppurating. To expose the hypotympanum it is sometimes necessary to shave down the tympanic end of the meatal floor, and this, as well as the removal of the facial ridge down to the level of the external semicircular canal requires considerable care. Chisels have the great disadvantage of cutting from without inwards. I have found the Richards round-nosed cutting curettes, which I saw him using in New York at the Manhattan Ear and Throat Hospital, of very great service. Richards states that "by holding the curette vertically, placing its nose upon the summit of the external semicircular canal, using the superior rim of the bone cavity as a fulcrum and working toward the tip, the ridge can be shaved down to the desired level with the utmost ease, and the danger of injuring the nerve is practically eliminated ; for the plane of action of the curette is either parallel to the course of the nerve, or else in the direction from it, and the cutting edge is (if the instrument be held vertically) slightly above the convexity of its nose. Even though the nose of the instrument rests upon an exposed nerve, that structure is practically insured from being cut across. Not only is the curette more effective here than the chisel, but, what is more important, it is immeasurably safer." For the same purpose, I have found a bone plane I devised of very great use ; it shaves off very thin slices of the bone, and cannot penetrate deeply so as to suddenly injure or divide the nerve, being made on the principle of the safety shaver.

Of the importance of cleanly removing the pillars of the aditus, and of the outer tympanic wall, I will say nothing, as these are too generally recognised to require any advocacy ; but it may not be out of place to emphasise the desirability of carefully curetting the tympanic end of the Eustachian tube, as here infected granulations are commonly present, and when neglected seriously interfere with the healing process. After curettement pure carbolic acid may be applied to the upper end of the tube.

It is questionable whether the plan of substituting for the daily packing of the mastoid cavity the use of spirit drops has not greater drawbacks than advantages, although by so doing the patient is saved considerable discomfort and pain. Granulations, I think, are more prone to bridge over and give subsequent trouble. I have found it possible to dispense with packing with gauze earlier than usual by packing the mastoid cavity, after cleansing and drying, with boracic acid and borax powder, and keeping it in place by simply packing the outer part of the meatus with gauze. It is then simple and painless to syringe out the boracic acid powder, cleanse the cavity, and repack in the same way as often as may be required.

*Discussion.*—Mr. J. LACY FIRTH remarked that he endeavoured to remove all the mucous membrane in the bony cavity to secure complete epidermisation, and that it was unusual to get such good hearing power as the patient shown possessed, and beforehand it was not possible to say what the effect of the operation upon the hearing capacity would be.

Fig. 1.
*Richards' Cutting Curette.*

Fig. 2.
*The Author's Safety Bone-shaver.*

## Figures and Tables

**Fig. 1. f1:**
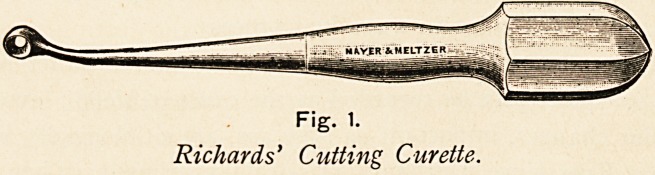


**Fig. 2. f2:**